# A bright triggered twin-photon source in the solid state

**DOI:** 10.1038/ncomms14870

**Published:** 2017-04-03

**Authors:** T. Heindel, A. Thoma, M. von Helversen, M. Schmidt, A. Schlehahn, M. Gschrey, P. Schnauber, J. -H. Schulze, A. Strittmatter, J. Beyer, S. Rodt, A. Carmele, A. Knorr, S. Reitzenstein

**Affiliations:** 1 Institut für Festkörperphysik, Technische Universität Berlin, Hardenbergstraße 36, 10623 Berlin, Germany; 2 Physikalisch-Technische Bundesanstalt, Abbestraße 2-12, 10587 Berlin, Germany; 3 Institut für Theoretische Physik, Technische Universität Berlin, Hardenbergstraße 36, 10623 Berlin, Germany

## Abstract

A non-classical light source emitting pairs of identical photons represents a versatile resource of interdisciplinary importance with applications in quantum optics and quantum biology. To date, photon twins have mostly been generated using parametric downconversion sources, relying on Poissonian number distributions, or atoms, exhibiting low emission rates. Here we propose and experimentally demonstrate the efficient, triggered generation of photon twins using the energy-degenerate biexciton–exciton radiative cascade of a single semiconductor quantum dot. Deterministically integrated within a microlens, this nanostructure emits highly correlated photon pairs, degenerate in energy and polarization, at a rate of up to (234±4) kHz. Furthermore, we verify a significant degree of photon indistinguishability and directly observe twin-photon emission by employing photon-number-resolving detectors, which enables the reconstruction of the emitted photon number distribution. Our work represents an important step towards the realization of efficient sources of twin-photon states on a fully scalable technology platform.

## Abstract (web summary)

Photon twins are important for interdisciplinary research fields using non-classical light, such as quantum biology. Here, Heindel *et al*. demonstrate that a single semiconductor quantum dot integrated into a microlens operates as an efficient photon-pair source.

Quantum light sources are key building blocks for future photonic technologies^1^,^2^. The underlying processes to create and control non-classical states of light are challenging tasks at the heart of quantum optics^3^,^4^,^5^,^6^,^7^. Aside from the mere spirit of research, pursuing deeper access to the quantum world, the related research is strongly driven by applications in the fields of communication^8^, information processing^9^ and metrology^10^. In this context, solid-state-based non-classical light emitters are of particular interest, due to the prospects of device integration and scalability. The engineering of quantum light sources emitting single photons has made great progress in recent years^11^. Close to ideal single-photon sources can nowadays be realized using semiconductor quantum dots (QDs) and emerging deterministic fabrication techniques significantly increase the device yield by embedding single, pre-selected quantum emitters within photonic microstructures^12^,^13^,^14^.

Compared with the huge progress made in the development of single-photon sources, the on-demand generation of more complex photonic states is still in its infancy. An interesting example for multipartite quantum light states are photon twins, that is pairs of temporally correlated photons with identical properties. Until now, twin photons were generated using nonlinear crystals^15^ or atomic systems^16^,^17^, both of which suffer from low photon emission rates and limited scalability. Integrated schemes using spontaneous parametric downconversion for the generation of photon twins have been demonstrated^18^, but still exhibit low efficiencies and rely on intrinsically non-deterministic emission processes. Semiconductor QDs, on the other hand, turned out to be excellent quantum emitters^19^,^20^,^21^, which can produce single-photon states with high efficiency under triggered optical^22^,^23^,^24^ as well as electrical^25^ excitation. Interestingly, they also allow for the generation of correlated photon pairs by exploiting the biexciton-exciton radiative cascade^26^. Here, two electron–hole pairs form the biexciton state, which radiatively decays under emission of two photons via the single exciton state to the ground state. So far, experiments exploiting the biexciton–exciton radiative cascade, which typically have been aimed at the generation of entanglement^27^,^28^, relied entirely on pairs of photons with different energies. The possibility to directly generate photon twins has remained elusive until now.

Here we propose and experimentally demonstrate an integrated source of photon twins, that is pairs of photons with identical energy and polarization, highly correlated in time. For this purpose, we use a QD exhibiting an energy degenerate biexciton–exciton radiative cascade integrated deterministically within a monolithic microlens fabricated by three-dimensional (3D) *in-situ* electron-beam lithography. Twin-photon emission of our quantum light source is studied and verified via polarization-resolved photon-correlation measurements. In addition, we verify a significant degree of photon indistinguishability in Hong–Ou–Mandel (HOM) -type two-photon interference (TPI) experiments. To directly observe the twin-photon emission of our source, we further employ a photon-number-resolving (PNR) detector, which enables us to reconstruct the photon number distribution emitted by the twin-photon source and to compare the result with a QD-based single-photons source. Combining our concept of twin-photon generation with resonant excitation schemes, we anticipate potential for the generation of close-to-ideal twin-photon states on a fully scalable technology platform.

## Results

### Concept of the deterministic twin-photon source

**Figure 1:**
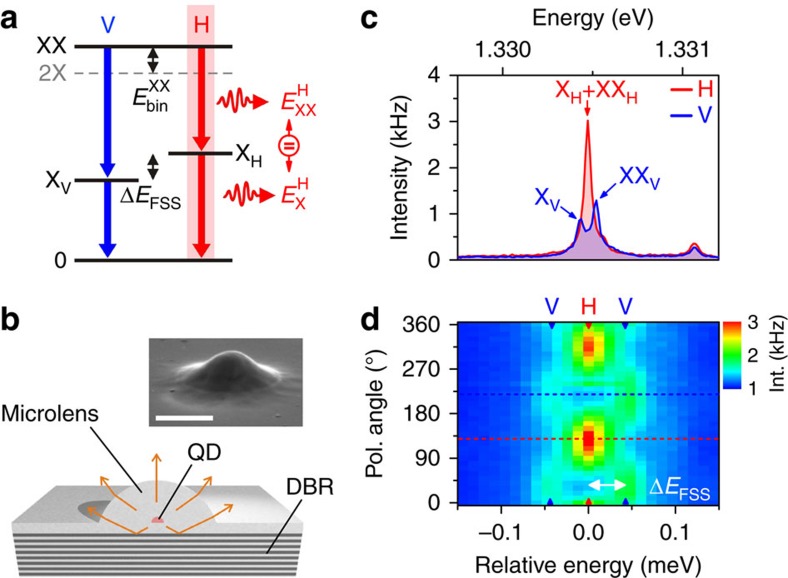
Concept of a deterministically integrated twin-photon source. (**a**) Energy level scheme of a radiative cascade involving the biexciton- (XX), exciton- (X) and ground- (0) state. For finite fine structure splitting Δ*E*_FSS_, the possible decay channels are linear-horizontally (H) and linear-vertically (V) polarized. In case of *E*_*X*_^H^=*E*_XX_^H^, the exciton fine structure splitting Δ*E*_FSS_ equals the biexciton binding energy *E*_bin_^XX^ and the photons within the H-polarized decay channel exhibit identical energy and polarization. (**b**) Illustration of our solid-state based quantum light source constituted of a single QD deterministically integrated within a monolithic microlens. The microlens design in combination with a lower distributed-Bragg reflector (DBR) allows for an enhanced photon collection efficiency of photons emitted by the QD. Inset: scanning electron microscopy image of a microlens (scale bar, 1 μm). (**c**) Spectrally resolved photoluminescence of a single-QD microlens for H- and V-polarization. For H-polarization, the superimposed emission of exciton and biexciton leads to an increased emission intensity compared to V-polarization. (**d**) Polarization-resolved emission spectra in a close up with relative energy scale. A quantitative analysis reveals Δ*E*_FSS=_|*E*_bin_^XX^|=(51±6) μeV. By selecting the H-polarized decay channel photon twins can be extracted. Dashed lines indicate the position of the spectra displayed in **c**.

The biexciton state of a QD is constituted of two bound electron-hole pairs. Owing to Coulomb and exchange interactions of the involved charge carriers, this state typically shows a finite binding energy *E*_bin_^XX^ with respect to the case of two unbound excitons, which is in case of the InGaAs/GaAs material system on the order of ∼1 meV^29^. The exciton state, on the other hand, consists of a single electron–hole pair and usually reveals a fine structure splitting Δ*E*_FSS_ on the order of ∼10 μeV^30^, which arises from anisotropic electron–hole exchange interaction. The resulting radiative cascade emits pairs of photons in two possible decay channels, one being linear-horizontally (H) and the other one linear-vertically (V) polarized. Owing to the energy scales of *E*_bin_^XX^ and Δ*E*_FSS_ mentioned above, this configuration leads to two doublets of orthogonally linearly polarized emission lines visible in the emission spectra of exciton and biexciton states, exhibiting spectrally distinguishable photons. In this work, we selected a QD featuring *E*_X_^H^=*E*_XX_^H^ (Fig. 1a), which is a direct consequence of Δ*E*_FSS_=|*E*_bin_^XX^| for the chosen radiative cascade. For this particular energy level alignment, one decay channel of the biexciton–exciton cascade reveals the emission of photon twins—a non-classical light state constituted of two temporally correlated photons with identical emission energy and polarization. The QD is deterministically integrated within a monolithic microlens (Fig. 1b) by means of 3D *in-situ* electron-beam lithography^31^, which provides enhanced photon collection efficiency for the twin-photon generation process (see Methods). Figure 1c shows photoluminescence spectra of the QD emission under above-bandgap (*λ*=850 nm) continuous-wave excitation for H- and V-polarization. In case of V-polarization, a doublet centred at 1.33047, eV is observed, where the low- and high-energy component can be attributed to the excitonic (X_V_) and biexcitonic (XX_V_) emission, respectively. Switching to H-polarization, a single, intense emission line can be observed at 1.33047, eV. This behaviour is analysed in more detail in Fig. 1d, depicting a polarization-resolved map of photoluminescence spectra. Exciton and biexciton exhibit a sinusoidal shift in energy with opposite phase; however, close to H-polarization their emission becomes superimposed, resulting in a distinct maximum of the emission intensity. A quantitative analysis of the spectra from Fig. 1d yields Δ*E*_FSS_=|*E*_bin_^XX^|=(51±6) μeV (see Supplementary Note 1 and Supplementary Fig. 1). The fact that we observe an antibinding biexciton state (*E*_XX_^V^>*E*_X_^V^) in this case, is indicative for a relatively small QD size^32^.

### Polarization-resolved photon correlations

**Figure 2:**
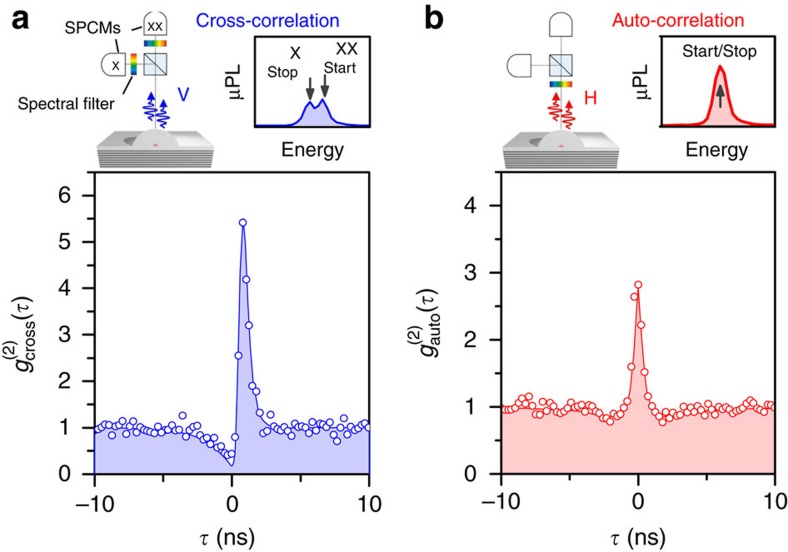
Polarization-resolved photon-correlations of photon pairs. (**a**) Photon cross-correlation histogram 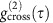 for the V-polarized decay channel, where biexciton and exciton emission are spectrally separated (*cf*. schematic of experiment and spectrum, where single-photon counting modules (SPCMs) are used for coincidence measurements). The strong bunching signature at *τ*>0 in combination with an antibunching at *τ*<0 proves the cascaded emission of biexciton-exciton photon pairs. (**b**) Photon auto-correlation histogram 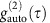 for the H-polarized decay channel of the biexciton-exciton cascade, where biexciton and exciton emission are superimposed (*cf*. schematic). The pronounced bunching at *τ*=0 with 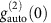
=2.85 indicates a high degree of two-photon correlations, due to the emission of photon twins. Solid curves in both panels are theoretical simulations based on a four-level master equation approach accounting for the experimental conditions.

The dynamics of this unique four-level system were studied via polarization-resolved photon-correlation measurements^33^. First, we address the correlations of the V-polarized cascade channel. In this case, exciton and biexciton photons are energetically separable using two spectrometers (schematic in Fig. 2a). Figure 2a displays the obtained cross-correlation coincidence histogram 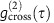, where biexciton photons started and exciton photons stopped the measurement. An asymmetric bunching effect is observed for positive delay times *τ*, owing to the cascaded emission of photon pairs within the same decay channel^34^. Next, the photon correlations of the H-polarized decay channel are investigated. Here, exciton and biexciton photons are energetically degenerate and temporal correlations can be probed via photon auto-correlation measurements using a single spectrometer (schematic in Fig. 2b). The corresponding coincidence histogram reveals a prominent bunching signature at zero delay and, due to the absence of time-ordering of the detected photons, a symmetric behaviour in *τ*. The pronounced bunching indicates a high degree of two-photon correlations, which proves that this unique biexciton–exciton radiative cascade serves as a source of photon twins. In addition, we detect clear antibunching at finite delay times (*τ*=±2 ns), signifying the non-classicality of the emitted light state. Our experimental observations agree quantitatively with a theoretical model (solid curves) based on a four-level rate equation approach (see Supplementary Note 2).

### Quantifying the source efficiency

**Figure 3:**
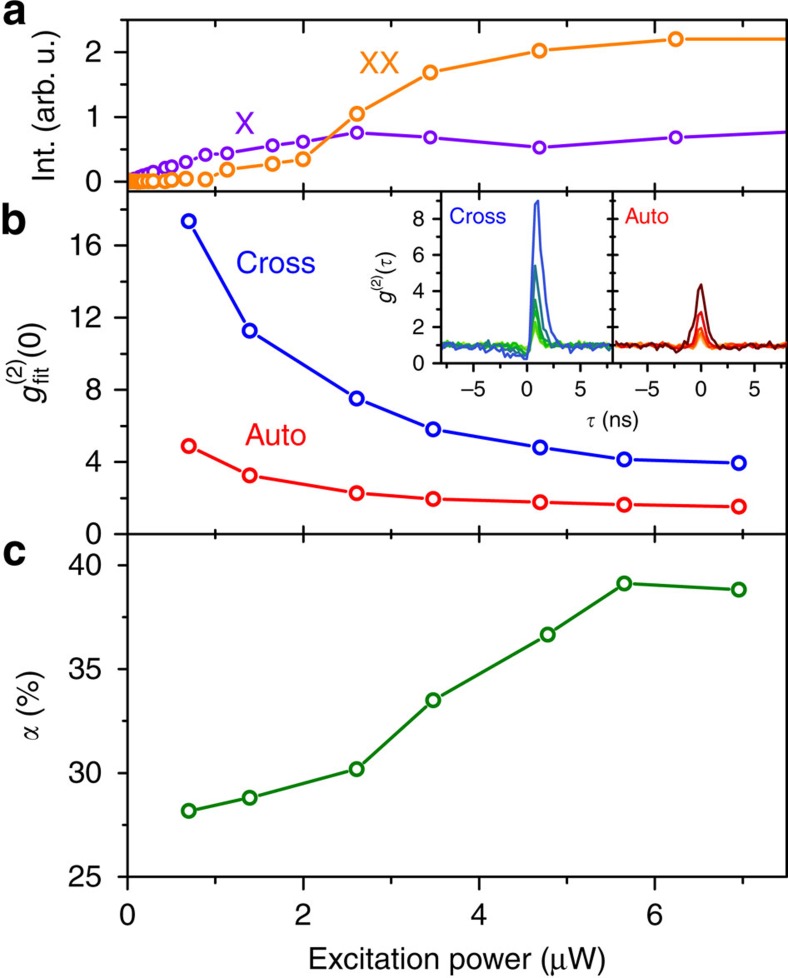
Excitation power dependence of twin-photon generation. (**a**) Integrated intensities of exciton and biexciton emission extracted for the V-polarized decay channel. The emission intensities of exciton and biexciton saturate at excitation powers of about 2 and 6 μW, respectively. (**b**) Bunching values 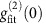 for auto- and cross-correlation resulting from a theoretical fit to the experimental data shown in the inset (taking into account the timing resolution of the setup). (**c**) Fraction of two-photon correlations due to twin-photon emission 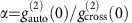 calculated from the bunching values in **b**.

The magnitude of the bunching in Fig. 2 itself, however, does not carry information about the probability of having a photon pair per excitation. In fact, the bunching value depends mainly on the occupation of the exciton level (inversely proportional), rather than on the biexciton occupation. For this reason and to quantify the efficiency of our source, we introduce the parameter 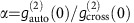, that is, the ratio of the bunching values in auto- and cross-correlation, respectively. The parameter *α* thereby corresponds to the fraction of two-photon correlations due to twin-photon emission, which naturally follows if one considers that the observable of our photon auto-correlation measurement on the H-polarized decay channel results from the superposition of a total of four different photon correlations (see Methods for details and Supplementary Note 2 for the explicit expressions of the observables). Hence, by comparing the measured cross- and auto-correlation traces, one obtains information about the efficiency of the twin-photon cascade. Figure 3a displays the excitation power dependencies of the integrated intensities of the biexciton and exciton emission (extracted in V-polarization). The corresponding bunching values 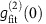 are depicted in Fig. 3b and result from a deconvolution of the measured auto- and cross-correlation traces (Fig. 3b, inset), by applying our theoretical model and taking into account the timing resolution of the setup. The extracted bunching magnitude reveals a monotonic drop with increasing excitation. This behaviour is typically observed in excitation-power dependent cross-correlation measurements^35^,^36^ and does not carry information on the twin-photon generation efficiency (as discussed above). However, the decrease of the bunching for photon twins (auto-correlation) is less pronounced compared to the distinguishable XX–X photon pairs (cross-correlation), which indicates a change in the generation efficiency of photon twins in the degenerate cascade channel. Figure 3c presents the respective ratio *α* calculated from Fig. 3b. With increasing excitation, the cascade efficiency steadily increases and reaches a maximum value of *α*=(39±3)%. From this, we can deduce the twin-photon emission rate (TPR) collected via the microscope objective to be (234±4) kHz (see Methods). This represents a significant improvement (by a factor of 5) compared with photon twins generated with atoms^17^. As the outcoupling of a photon twin depends quadratically on the photon-extraction efficiency of the microlens, we anticipate further improved TPRs of ∼1.3–2.1 MHz using anti-reflection coatings^37^ or a bottom gold mirror^31^ (assuming photon extraction efficiencies of 50–80% and excitation at *λ*=850 nm).

### Triggered generation of indistinguishable photon twins

**Figure 4:**
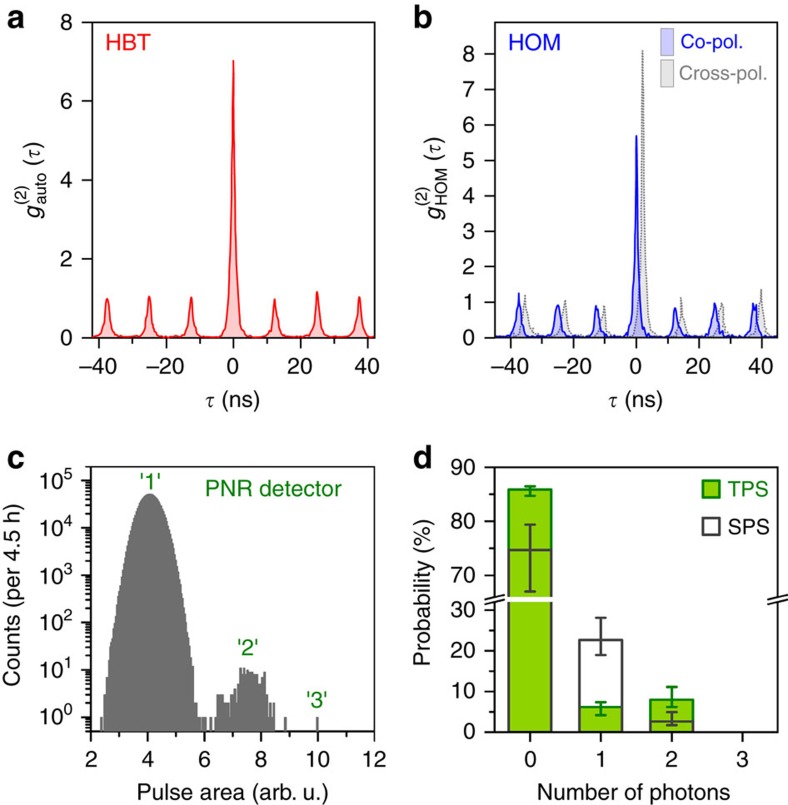
Triggered generation of photon twins. (**a**) Measured photon auto-correlation histogram 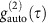 of photon twins under pulsed above-band excitation at low excitation power (*P*=87 nW). A bunching of 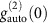
=*A*_0_/*A*=5.1 is observed, where *A*_0_ and *A* correspond to the zero-delay peak and the peaks at finite *τ*. (**b**) HOM TPI experiment using photon twins under pulsed *p*-shell excitation. Measured histograms of 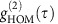 for co-polarized (solid line) and cross-polarized (dashed line) configuration using a symmetric Mach–Zehnder interferometer (data in cross-polarization shifted by +2 ns for clarity). (**c**) Direct detection of photon twins using a PNR detection system based on a TES. The histogram shows the pulse-area-distribution of photon detection events from the twin-photon cascade, where the labeled peaks correspond to the detection of one (‘1'), two (‘2') or three (‘3') photons. (**d**) Reconstructed photon number distribution of the twin-photon source (TPS), deduced from the PNR measurement in **c** by taking into account the total losses of the experimental setup. Results obtained for a quantum dot single-photon source (SPS) are displayed for comparison. The error bars result from the uncertainties of the setup efficiency and the photon extraction efficiency of the microlens.

To operate our quantum light source as a two-photon gun, we applied pulsed excitation in the following. Figure 4a displays the auto-correlation histogram of the energy-degenerate decay channel (H-polarization) under above-band (*λ*=850 nm) pulsed excitation at a repetition rate of *f=*80 MHz. The observed strong bunching effect proves the predominance of two-photon correlations due to pulsed twin-photon emission. Next, pulsed twin-photon emission was utilized to test the indistinguishability of both photons emitted within the H-polarized decay channel, by means of HOM-type TPI experiments (see Methods). For this purpose, we excited the QD into its *p*-shell (*λ*=904.5 nm) and sent the triggered photon twins into a symmetric Mach–Zehnder interferometer, where a half-wave plate within one interferometer arm allows for switching between the co- and cross-polarized measurement configuration. Figure 4b presents the TPI histograms 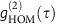 for both measurement configurations. A clear reduction in coincidences is observed at *τ*=0 for co-polarized measurement configuration, proving the interference of photons emitted by the cascade. Considering the bunching values 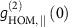 and 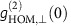 extracted from the coincidence peak-area ratios yields a visibility of TPI of 
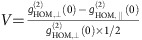
 =(56±9)% (see Methods). Limiting factors for the observed indistinguishability are discussed in Supplementary Note 3 and can be overcome by applying strictly resonant excitation schemes via simultaneous one- and two-photon excitation (TPE) or by exploiting cavity effects.

To directly detect the generated twin-photon state, we employed a state-of-the-art PNR detection system based on a transition-edge sensor (TES; see Methods). Figure 4c presents the measured photon number distribution of the H-polarized decay channel, triggered by a pulsed diode laser at a repetition rate of 1 MHz (*λ*=661 nm). Detection events corresponding to photon twins (‘2') are clearly identified and well separated from the single-photon detection events. Furthermore, we can derive the photon number distribution emitted by our source, taking into account the total losses of the experimental setup (see Methods). As illustrated in Fig. 4d, the probability for twin-photon emission is *p*_twin_=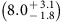
% in case of the degenerate cascade channel, being noticeably larger than the probability for single-photon emission 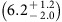
%. As expected, these probabilities completely change in case of a single-photon source, showing 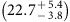
% single-photon emission and only 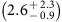
% photon pair emission. In both cases, the relatively large contribution of the vacuum state is partly related to the less efficient excitation by the pulsed diode laser (661 nm) used for the PNR experiment. In case of the twin-photon source, additionally the population of the V-polarized decay channel is not detected due to the selection of the H-polarization, which artificially increases the contribution of the vacuum state. From the twin-photon emission probability *p*_twin_, we can further deduce the triggered TPR collected via the microscope objective to be 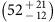 kHz (see Methods). Using a more efficient excitation scheme at a wavelength of 850 nm would thereby readily allow for a threefold enhancement of the TPR.

## Discussion

Although the probability to observe the energy level alignment presented in our work for an as-grown QD is relatively low (<1%), the device yield for twin-photon sources can be significantly improved in the future. Applying an advanced *in-situ* fabrication technique involving a detailed precharacterization^38^, QDs with particular small biexciton binding energy can be pre-selected in advance. Beyond that, even a fully scalable device concept is within reach employing existing technologies. For instance, using external strain-tuning via piezo-actutators^39^, the biexciton binding energy can be precisely adjusted, which would substantially increase the device yield. Furthermore, our scheme can also be extended towards electrical control or current injection via electrical gates^40^.

In addition, resonant excitation, which is typically applied for the coherent excitation of single excitonic states, would be particularly interesting in the presented case of a degenerate biexciton–exciton cascade, where the laser coherently drives two quantum emitters: The biexciton state via TPE^41^ and the exciton state via one-photon excitation, at which the X_H_-level is congruent with the virtual intermediate level of the TPE process. In such case, it could be possible to achieve a biexciton occupation close to unity, which would greatly enhance the parameter *α*, that is, the fraction of two-photon correlations due to twin-photon emission and hence boost the efficiency of the twin-photon source. As the TPE depends quadratically on the excitation power, this can be tested in the regime of strong pumping (requiring a multiple-*π*-pulse), at which TPE processes dominate the single-photon excitation of the exciton level. It might be even possible in this regime, to produce coherently excited two-photon states, which do not reveal the intrinsic time ordering of the cascade, as recently observed in experiments on the dressing of the biexciton state in QDs^42^. This could finally lead to the realization of efficient sources of close-to-ideal two-photon Fock-states |2〉 on a fully scalable technology platform.

In summary, we introduced an attractive type of integrated twin-photon source based on a QD deterministically integrated within a monolithic microlens. Triggered generation of photon pairs with the same energy and polarization becomes possible by utilizing a biexciton–exciton radiative cascade, where the biexciton binding energy equals the fine structure splitting of the bright exciton. We observe strong temporal correlations of the photon twins in auto-correlation measurements, resulting in a pronounced symmetric bunching peak. Further, by comparing the measured cross- and auto-correlation traces, we are able to determine the efficiency of the twin-photon cascade and demonstrate a TPR of (234±4) kHz. In addition, we employ a PNR detector to directly verify twin-photon emission and to reconstruct the photon number distribution emitted by the quantum emitter. The proposed quantum light source is very attractive for novel quantum optics experiments in the fields of quantum-optical spectroscopy^43^,^44^,^45^ or quantum biology^46^.

## Methods

### Sample

The QD sample used for our experiments was grown by metal-organic chemical vapour deposition on a GaAs (001) substrate. Self-organized InGaAs QDs are deposited above a lower distributed Bragg reflector constituted of 23 alternating *λ*/4-thick bilayers of AlGaAs/GaAs. On top of the QD layer, a 400 nm**-**thick GaAs capping layer provides the material for the subsequent microlens fabrication. Deterministic single-QD microlenses were processed via 3D *in-situ* electron-beam lithography based on low-temperature cathodoluminescence spectroscopy^31^. Shallow hemispheric-section-type microlenses with heights of 400 nm and base widths of 2.4 μm were chosen, allowing for a photon extraction efficiency of up to 29% (ref. 47).

### Experimental setup

For the micro-photoluminescence investigations, the sample is mounted onto the cold-finger of a liquid-Helium-flow cryostat and held at a temperature of *T*=6 K. The QD microlens is optically excited using a wavelength tunable Ti:sapphire laser operating in continuous-wave or pulsed picosecond mode (*f*=80 MHz). Photoluminescence is collected via a microscope objective with a numerical aperture of 0.4 serving as first lens of the detection system. The micro-photoluminescence signal is spectrally analysed using a grating spectrometer with an attached charge-coupled device camera enabling a spectral resolution of 25 μeV. Two-photon emission of the deterministic QD microlens is further studied via polarization-resolved photon-correlation experiments. In case of photon auto-correlation measurements, the superimposed exciton and biexciton emission (H-polarization) is spectrally selected using a single monochromator and analysed using a fibre-based Hanbury–Brown and Twiss setup containing a 50:50 multi-mode beamsplitter. For photon cross-correlation measurements, the spectrally distinguishable exciton and biexciton emission (V-polarization) is spatially separated using two monochromators. In both cases (auto- and cross-correlation), coincidence measurements are performed using two fibre-coupled silicon-based single-photon counting modules (SPCMs) with an overall timing resolution of 350 ps in combination with time-correlated single-photon counting electronics with 4 ps time-bin width. To determine the efficiency of our twin-photon source from the detected count rates at the SPCMs, we measured the collection efficiency ɛ of our experimental setup to be (0.95±0.05)% following ref. 31. The indistinguishability of photons from the emitted biexciton–exciton pair is studied by means of HOM-type TPI measurements via a Mach–Zehnder interferometer based on polarization maintaining fibres. A half-wave plate allows to switch the polarization of photons in one of the interferometer arms, either being co- or cross-polarized with respect to photons in the other arm. The Mach–Zehnder interferometer in this work (in contrast to ref. 31) was chosen symmetric with respect to the arm length, to account for a negligible temporal delay between photon twins.

### Twin-photon generation efficiency

To quantify the efficiency of our twin-photon source, we introduced the parameter 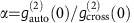, describing the fraction of two-photon correlations due to twin-photon emission. This can be explained by considering the actual observables of our measurement in Fig. 2. In case of the cross-correlation, each start and stop trigger of the coincidence measurement can be attributed to the distinct detection of one XX and one X photon. The corresponding observable of this cross-correlation measurement can be expressed (for *τ*≥0) by 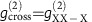. In case of the auto-correlation measurement on the degenerate H-polarized decay path, XX and X photons can be detected at both detectors. Thus, the distinct time order is lost and the correlation 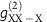 from above is superimposed by the time-inverted correlation 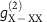. In addition, also the true auto-correlations 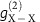 and 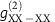 of exciton and biexciton, respectively, have to be taken into account. The complete two-photon correlation 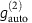 for H-polarization thus reads (*τ*≥0):


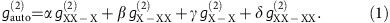


At zero delay-time (*τ*=0) only the term *g*^(2)^_XX−X_ on the right hand side of equation (1) has a non-zero contribution 
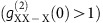
, while in all other cases the QD has to be refilled with either one or two electron–hole pairs before stop-photon detection and hence 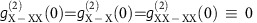. These considerations result in the expression 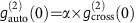 and *α* is expected to be ¼ in case of equally distributed probabilities for all possible correlations. It follows, that one observes a preferred twin-photon emission, that is enhanced probability for the correlation 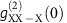, if *α*>¼ (while *α*+*β*+*γ*+*δ*=1). Thus, by introducing the parameter *α* we are able to quantify the efficiency of the cascade, despite the excitation-dependent exciton occupation discussed in previous reports^34^,^35^.

### Twin-photon emission rate from continuous-wave experiments

To deduce the twin-photon emission rate (collected via the microscope objective) from the experimentally determined *α*, one must take into account the measured count rates *n*_SPCM_=103 kHz at the SPCMs, the setup efficiency *ɛ*=(0.95±0.05)% for photons emitted into the first lens and the photon extraction efficiency *η*=(9±1)% of the microlens (both, *ɛ* and *η* were measured independently according to ref. 31). At this point, we assume that the detected rate of photon twins at the SPCMs is negligible, such that the photon stream consists of single photons. These parameters at hand, we can calculate the photon rate emitted by the QD. According to our measurement *α*=39% of the two-photon correlations originate from the emission of photons twins (XX−X). The two-photon coincidences resulting from the remaining contributions (weighted by *β*+*γ*+*δ*), however, result from photons in different excitation cycles (X−X, XX−XX, X−XX) and have to be counted as single photons. To be consistent and to calculate back to the contribution of single photon events one has to count them twice. This results in a probability of 24% for detecting a photon twin and 76% for the detection of single photons. With these values, we can calculate the twin-photon rate (TPR) collected via the microscope objective: 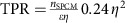
=(234±4) kHz, where the quadratic dependence on *η* was taken into account for photon twins.

### Two-photon interference visibility

To extract the TPI visibility from measured 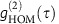 traces, we first determined the peak area ratio *g*^(2)^_HOM_(0)=*A*_0_/*A*, where *A*_0_ corresponds to the area of the zero-delay peak and *A* is the mean area of the peaks at *τ* ≠ 0. Even in the case of perfect indistinguishability between X and XX photons of the photon twins, one expects a finite contrast between the measurements in co- and cross-polarized configuration according to 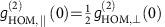. The respective coincidences in co-polarized configuration arise from the fact, that in 50% of all cases both photons of the exciton–biexciton pair will take the same path within the interferometer. Hence, they enter the second beam splitter at the same entrance port and thus cannot lead to TPI. Consequently, the TPI visibility has to be renormalized by a factor of 2 compared with the standard formula in ref. 48 according to 
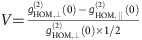
.

### Photon-number-resolving measurements

For the PNR experiments, we employed a detection system based on a fibre-coupled TES operated in a cryogenic environment. The TES thereby acts as a highly sensitive calorimeter, which is able to detect smallest amounts of energy dissipated during photon absorption^49^. The detector is voltage-biased to heat up the electron system within its superconducting-to-normal-conducting transition (∼152 mK) in the self-calibrated region. The implemented circuit allows for detecting a temperature increase, which causes a change in the resistance, ultimately leading to a detectable change in current. The latter is measured via an inductively coupled two-stage dc-superconducting quantum interference device^50^. For optimized absorption in the near infrared, the TES is embedded within a dielectric cavity^51^, resulting in a detection efficiency of ∼84% in the spectral region of interest for the detector used here. The TES/superconducting quantum interference device detector unit is mounted onto the cold stage of an adiabatic demagnetization refrigerator stabilized at 100 mK.

For the PNR experiment, the emission of our twin-photon source is triggered by a pulsed diode laser (pulse duration ∼80 ps) at a repetition rate of 1 MHz (*λ*=661 nm). The lower repetition rate is required in case of the PNR experiments, due to the relatively long thermal recovery time (∼1 μs) after photon detection. The emission of the H-polarized decay path is spectrally filtered (bandwidth: 120 μeV) and coupled to the TES, using a single-mode fibre (Thorlabs 780HP) positioned right above the detector chip. To reduce contributions of background counts as far as possible, we first triggered our experiment with the detection of photons falling within a 220 ns-wide time window in succession of the laser trigger (taking into account the signal propagation time). This trigger mode enabled us to reduce the background counts down to ∼3.6 twins per hour and ∼36 singles per hour, caused by spurious detection events of ambient light photons entering the refrigerator via the optical fibre. Within a measurement period of 4.5 h, we detect a total of 215 photon twins emitted by our QD source and we extract a twin-to-single photon ratio of ‘2/1'=(1.81±0.05) × 10^−4^ from the recorded histogram shown in Fig. 4c. In addition, we determined the vacuum contribution by a second measurement, at which the laser sync output was used as a trigger. We determine a ratio of single-photon detection events to vacuum contribution of ‘1/0'=1.1 × 10^−4^ within an acquisition time of 18 min. To deduce the photon number distribution emitted by the QD from the detected ratios ‘2/1' and ‘1/0', we take a binomial distribution into account^52^, where the number of independent Bernoulli trials is given by the photon number *n*=0, 1, 2 according to the detection of zero, one and two photons, and the success probability of each Bernoulli trial is the product *ɛ*_PNR_ × *η*=0.0504% of the setup transmission *ɛ*_PNR_=(0.56±0.04)% and the photon extraction efficiency of our microlens *η*=(9±1)%. From the extracted probability for twin-photon emission *p*_twin_=8.0% we are able to calculate the triggered TPR collected via the microscope objective by 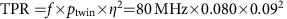
=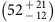 kHz, by assuming an excitation rate of *f*=80 MHz. The complete procedure described above (PNR experiments and data analysis) was additionally carried out for a QD single-photon source, for a better comparison. The reconstructed photon number distributions resulting from these experiments are illustrated in Fig. 4d.

### Data availability

The data that support the findings of this study are available from the corresponding author upon request.

## Additional information

**How to cite this article:** Heindel, T. *et al*. A bright triggered twin-photon source in the solid state. *Nat. Commun.* **8,** 14870 doi: 10.1038/ncomms14870 (2017).

**Publisher's note**: Springer Nature remains neutral with regard to jurisdictional claims in published maps and institutional affiliations.

## Supplementary Material

Supplementary Information Supplementary Figure, Supplementary Notes and Supplementary References

Peer Review File
