# Supplementary material for: A bright triggered twin-photon source in the solid state
Source: Nat Commun. 2017 Apr 3;8:14870. doi: 10.1038/ncomms14870 (PMC5382261; doi:10.1038/ncomms14870)
Supplement: Supplementary Information — Supplementary Figure, Supplementary Notes and Supplementary References [file ncomms14870-s1.pdf]

Supplementary Note 1: Polarization-resolved measurements

For a quantitative analysis of the quantum dot's (QD's) bright exciton fine-structure splitting ΔE_{FSS}, we applied fits to the spectra shown in Fig. 1d of the main article. We used four Lorentzian profiles corresponding to the linear-horizontally (H) and linear-vertically (V) -polarized components of the exciton- (X_H/X_V) and biexciton- (XX_H/XX_V) emission, respectively. For carrying out the fits we made the following reasonable assumptions: Firstly, both excitonic and both biexcitonic components are assumed to have the same spectral linewidth γ: $\gamma_{X,H} = \gamma_{X,V}$ and $\gamma_{XX,H} = \gamma_{XX,V}$. Secondly, the energetic splitting of the excitonic components equals the splitting of the biexcitonic components: $\Delta E_X^{H-V} = \Delta E_{XX}^{H-V} = \Delta E_{FSS}$. Additionally, the ratio of the integrated intensities of exciton and biexciton is the same for both polarizations: $I_{X,H}/I_{XX,H} = I_{X,V}/I_{XX,V}$. The resulting relative spectral positions of the emission lines extracted from the fits to a total of 36 spectra are depicted in Supplementary Figure 1a in a histogram. For clarity, the original polarization-resolved spectral map is displayed in Supplementary Figure 1b. The histograms reveal mean relative spectral positions of $\Delta E_{X,H} = (1.9 \pm 3.7)$ μeV and $\Delta E_{XX,H} = (-1.9 \pm 2.1)$ μeV for the H-polarized exciton and biexciton component, respectively, which coincide within their standard deviation. Hence, the photon twins emitted in the H-polarized decay channel can be considered degenerate in energy and polarization. Further, taking into account the relative spectral positions of $\Delta E_{X,V}$ and $\Delta E_{XX,V}$ for the V-polarized decay channel, we can extract a fine-structure splitting of $\Delta E_{FSS} = (51 \pm 6)$ μeV.

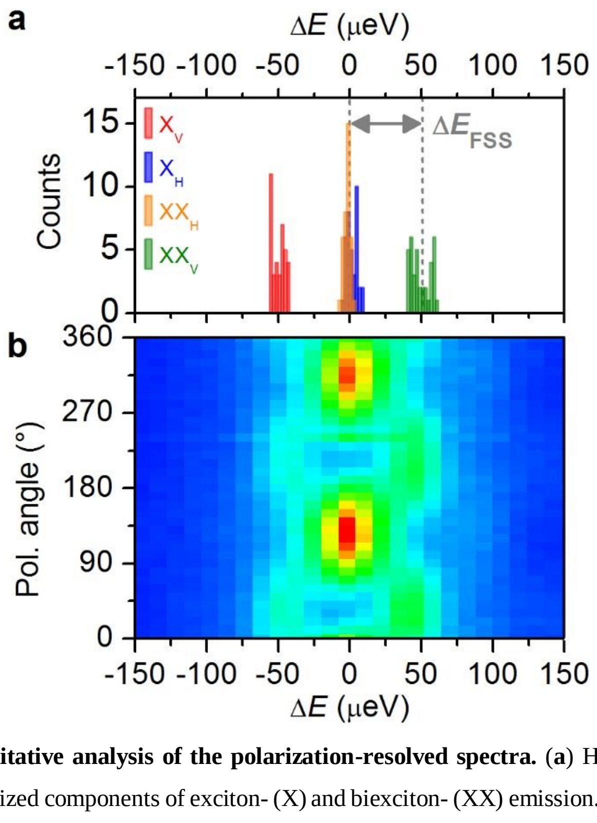

Supplementary Figure 1 | Quantitative analysis of the polarization-resolved spectra. (**a**) Histogram showing the relative spectral positions of the four H- and V-polarized components of exciton- (X) and biexciton- (XX) emission. The corresponding data were extracted from a total of 36 spectra displayed in the polarization-resolved spectral map in (**b**).

Supplementary Note 2: Rate equation model

For a theoretical description of our twin-photon source, we model the QD as a four-level system constituted of a ground-state $|\mathrm{G}\rangle$, two bright exciton states $|\mathrm{H}\rangle$ and $|\mathrm{V}\rangle$, and a biexciton state $|\mathrm{B}\rangle$. The dynamics of the excitonic levels can thus be expressed by the following rate equations for the state-occupation probabilities ρ:

$$\frac{d}{dt}\rho_{\mathrm{BB}} = -2\Gamma_{\mathrm{B}}\rho_{\mathrm{BB}} + P_{\mathrm{B}}(\rho_{\mathrm{VV}} + \rho_{\mathrm{HH}}) \quad (1)$$

$$\frac{d}{dt}\rho_{\mathrm{HH}} = -\Gamma_{\mathrm{X}}\rho_{\mathrm{HH}} + \Gamma_{\mathrm{B}}\rho_{\mathrm{BB}} - P_{\mathrm{B}}\rho_{\mathrm{BB}} + P_{\mathrm{X}}\rho_{\mathrm{GG}} \quad (2)$$

$$\frac{d}{dt}\rho_{\mathrm{VV}} = -\Gamma_{\mathrm{X}}\rho_{\mathrm{VV}} + \Gamma_{\mathrm{B}}\rho_{\mathrm{BB}} - P_{\mathrm{B}}\rho_{\mathrm{BB}} + P_{\mathrm{X}}\rho_{\mathrm{GG}} \quad (3)$$

$$\frac{d}{dt}\rho_{\mathrm{GG}} = -2P_{\mathrm{X}}\rho_{\mathrm{GG}} + \Gamma_{\mathrm{X}}(\rho_{\mathrm{HH}} + \rho_{\mathrm{VV}})\,, \quad (4)$$

where Γ_{B} (P_{B}) and Γ_{X} (P_{X}) correspond to the decay (pump) rates of the biexciton state and both exciton states. To connect the dynamics to the measurement, we assume within the far-field approximation that the photon detection events are proportional to dipole excitations: $c_i^\dagger \equiv \sigma_{\mathrm{B}i} + \sigma_{i\mathrm{G}}$, with i = H,V. Using this relation and the rate equations above (Supplementary Equation (1) to (4)), we can calculate the two-photon correlations $g^{(2)}(\tau \geq 0)$ in Equation 1 of the Methods section in the article using the quantum regression theorem[1]:

$$g^{(2)}_{\mathrm{XX-X}} = \frac{\langle\sigma_{\mathrm{BH}}(0)\sigma_{\mathrm{HG}}(\tau)\sigma_{\mathrm{GH}}(\tau)\sigma_{\mathrm{HB}}(0)\rangle}{\langle\sigma_{\mathrm{BH}}\sigma_{\mathrm{HB}}\rangle\langle\sigma_{\mathrm{HG}}\sigma_{\mathrm{GH}}\rangle}$$
$$= \frac{N\alpha}{4\alpha P^3\Gamma_{\mathrm{B}}}\left(\beta_1 + \beta_2 e^{-(\Gamma_{\mathrm{X}}+P)\tau} + \beta_3 e^{-\left(0.5\Gamma_{\mathrm{X}}+\Gamma_{\mathrm{B}}+1.5P-0.5\sqrt{\alpha}\right)\tau} + \beta_4 e^{-\left(0.5\Gamma_{\mathrm{X}}+\Gamma_{\mathrm{B}}+1.5P+0.5\sqrt{\alpha}\right)\tau}\right) \quad (5)$$

$$g^{(2)}_{\mathrm{X-XX}} = \frac{\langle\sigma_{\mathrm{HG}}(0)\sigma_{\mathrm{BH}}(\tau)\sigma_{\mathrm{HB}}(\tau)\sigma_{\mathrm{GH}}(0)\rangle}{\langle\sigma_{\mathrm{BH}}\sigma_{\mathrm{HB}}\rangle\langle\sigma_{\mathrm{HG}}\sigma_{\mathrm{GH}}\rangle}$$
$$= \frac{e^{-(\Gamma_{\mathrm{X}}+2\Gamma_{\mathrm{B}}+3P+\sqrt{\alpha})\frac{\tau}{2}}}{2}\left(\frac{\left(1-e^{\tau\sqrt{\alpha}}\right)(\Gamma_{\mathrm{X}}+2\Gamma_{\mathrm{B}}+3P)}{\sqrt{\alpha}} - 1 - e^{\tau\sqrt{\alpha}}\right) + 1 \quad (6)$$

$$g^{(2)}_{\mathrm{X-X}} = \frac{\langle\sigma_{\mathrm{HG}}(0)\sigma_{\mathrm{HG}}(\tau)\sigma_{\mathrm{GH}}(\tau)\sigma_{\mathrm{GH}}(0)\rangle}{\langle\sigma_{\mathrm{HG}}\sigma_{\mathrm{GH}}\rangle^2}$$
$$= \frac{e^{-(\Gamma_{\mathrm{X}}+2\Gamma_{\mathrm{B}}+3P+\sqrt{\alpha})\frac{\tau}{2}}}{2\Gamma_{\mathrm{B}}\sqrt{\alpha}}\left(-\Gamma_{\mathrm{B}}\sqrt{\alpha}\left(1+e^{\tau\sqrt{\alpha}}\right) - \left(1-e^{\tau\sqrt{\alpha}}\right)\left((\Gamma_{\mathrm{X}}-2\Gamma_{\mathrm{B}})\Gamma_{\mathrm{B}} + \Gamma_{\mathrm{B}}P + P^2\right)\right) + 1 \quad (7)$$

$$g^{(2)}_{\mathrm{XX-XX}} = \frac{\langle\sigma_{\mathrm{BH}}(0)\sigma_{\mathrm{BH}}(\tau)\sigma_{\mathrm{HB}}(\tau)\sigma_{\mathrm{HB}}(0)\rangle}{\langle\sigma_{\mathrm{BH}}\sigma_{\mathrm{HB}}\rangle^2}$$
$$= \frac{e^{-(\Gamma_{\mathrm{X}}+2\Gamma_{\mathrm{B}}+3P+\sqrt{\alpha})\frac{\tau}{2}}}{2P\sqrt{\alpha}}\left(\left(1-e^{\tau\sqrt{\alpha}}\right)(\Gamma_{\mathrm{X}}+P)(-2\Gamma_{\mathrm{B}}+P) - P\sqrt{\alpha}\left(1+e^{\tau\sqrt{\alpha}}\right)\right) + 1 \quad (8)$$

The following abbreviations were introduced for clarity:

$$P = P_{\mathrm{X}} = P_{\mathrm{B}}$$
$$N = \Gamma_{\mathrm{X}}\Gamma_{\mathrm{B}} + 2P\Gamma_{\mathrm{B}} + P^2$$
$$\alpha = (\Gamma_{\mathrm{X}} - 2\Gamma_{\mathrm{B}})^2 + 6\Gamma_{\mathrm{X}}P - 4\Gamma_{\mathrm{B}}P + P^2$$
$$\beta_1 = 4\Gamma_{\mathrm{B}}P\alpha$$
$$\beta_2 = 2\alpha N$$

$$\beta_3 = \Gamma_X^3\Gamma_B - P^2(2\Gamma_B - P)\left(-2\Gamma_B + P + \sqrt{\alpha}\right) + \Gamma_X^2\left(-4\Gamma_B^2 + 6\Gamma_B + P^2 - \Gamma_B\sqrt{\alpha}\right)$$
$$+ \Gamma_X\left(4\Gamma_B^3 + P^2\left(6P - \sqrt{\alpha}\right) + 2\Gamma_B^2\left(-2P + \sqrt{\alpha}\right) - 3\Gamma_B P\left(P + \sqrt{\alpha}\right)\right)$$
$$\beta_4 = \Gamma_X^3\Gamma_B + P^2(2\Gamma_B - P)\left(2\Gamma_B - P + \sqrt{\alpha}\right) + \Gamma_X^2\left(-4\Gamma_B^2 + 6\Gamma_B P + P^2 + \Gamma_B\sqrt{\alpha}\right)$$
$$+ \Gamma_X\left(4\Gamma_B^3 + P^2\left(6P + \sqrt{\alpha}\right) + 2\Gamma_B^2\left(-2P + \sqrt{\alpha}\right) + 3\Gamma_B P\left(-P + \sqrt{\alpha}\right)\right)$$
$$Z = 4\alpha N$$
$$\langle\sigma_{BH}\sigma_{HB}\rangle = \frac{P^2}{N} = a$$
$$\langle\sigma_{HG}\sigma_{GH}\rangle = \frac{P\Gamma_B}{N}$$

In case of the photon cross-correlation on spectrally separable biexciton-exciton photons (see article, Fig. 2a), the correlations $g^{(2)}_{X-XX}$ and $g^{(2)}_{XX-X}$ (Supplementary Equations (6) and (5)) must be considered for negative and positive temporal delay τ, respectively. In case of the photon auto-correlation on spectrally degenerate biexciton-exciton photons (see main article, Fig. 2b), all four correlations defined in the Supplementary Equations (5) to (8) get superimposed. To account for the timing resolution of the experimental setup, the theoretical correlation functions are convoluted with a Gaussian of 350 ps full-width at half maximum. Finally, the experimentally determined two-photon correlations in Fig. 2a and b as well as the inset of Fig. 3b are fitted using the derived model.

Supplementary Note 3: Indistinguishability of photon twins

For the two-photon interference visibility of photon twins observed in our experiment (Fig. 4b) the following contributions can be considered: Firstly, pure dephasing in terms of an inhomogeneous spectral broadening of the QD emission results in a reduced wavepacket overlap[2]. A method to measure the corresponding time scale of spectral diffusion has been introduced very recently[3] and can be encountered in the future by optimized QD growth[4] or electrically controlled microlens devices[5] to reduce electric field noise[6]. Secondly, in the case of interfering photons from the XX-X radiative cascade, exciton and biexciton state have different radiative lifetimes, independently measured to be $\tau_X = (1.77 \pm 0.05)$ ns and $\tau_{XX} = (0.95 \pm 0.08)$ ns, and the radiative decay of the biexciton introduces an additional time jitter between the X and XX photons. This issue can be encountered in the future by utilizing cavity effects to reduce the radiative lifetime via the Purcell effect or to induce spontaneous two-photon emission[7]. Additionally, strictly resonant excitation can be applied in the future, which eliminates any residual time jitter due to charge carrier relaxation.

Supplementary References:

1. Loudon, R. *The Quantum Theory of Light* 3rd edn. (Oxford University Press, 2000).

2. Santori, C., Fattal, D., Pelton, M., Vučković, J., Solomon, G. S. & Yamamoto, Y., Indistinguishable photons from a single-photon device. *Nature* **419**, 594-597 (2002).

3. Thoma, A. *et al.*, Exploring Dephasing of a Solid-State Quantum Emitter via Time- and Temperature-Dependent Hong-Ou-Mandel Experiments. *Phys. Rev. Lett.* **116**, 033601 (2016).

4. Wang, H. *et al.*, Near Transform-Limited Single Photons from an Efficient Solid-State Quantum Emitter. *Phys. Rev. Lett.* **116**, 213601 (2016).
5. Schlehahn, A. *et al.*, Generating single photons at gigahertz modulation-speed using electrically controlled quantum dot microlenses. *Appl. Phys. Lett.* **108**, 021104 (2016).
6. Somaschi, N. *et al.*, Near-optimal single-photon sources in the solid state. *Nat. Photon.* **10**, 340-345 (2016).
7. Ota, Y., Iwamoto, S., Kumagai, N. & Arakawa, Y., Spontaneous Two-Photon Emission from a Single Quantum Dot. *Phys. Rev. Lett.* **107**, 2033602 (2011).
