# Supplementary material for: A bright triggered twin-photon source in the solid state
Source: Nat Commun. 2017 Apr 3;8:14870. doi: 10.1038/ncomms14870 (PMC5382261; doi:10.1038/ncomms14870)
Supplement: Peer Review File [file ncomms14870-s2.pdf]

Editorial Note: this manuscript has been previously reviewed at another journal that is not operating a transparent peer review scheme. This document only contains reviewer comments and rebuttal letters for versions considered at Nature Communications.
